# Characterizing microcycles’ workload when combining two days structure within single training sessions during congested fixtures in an elite male soccer team

**DOI:** 10.5114/biolsport.2024.132992

**Published:** 2024-03-18

**Authors:** Antonio Gómez-Díaz, Fábio Yuzo Nakamura, Pedro Menezes, João Barreira, Pedro Figueiredo, Diogo Coutinho

**Affiliations:** 1 Spanish Football Federation (RFEF), Madrid, Spain; 2 Department of Sports Sciences and Physical Education, University of Maia, Maia, Portugal; 3 Research Center in Sports Sciences, Health Sciences and Human Development, CIDESD, 5000-801 Vila Real, Portugal; 4 Clube Regatas do Flamengo, Rio de Janeiro, Brazil; 5 Physical Education Department, College of Education, United Arab Emirates University, Al Ain, United Arab Emirates; 6 Portugal Football School, Portuguese Football Federation, Oeiras, Portugal

**Keywords:** Training Sessions, Team Sports, External Load, Playing Positions, Football

## Abstract

This study aimed to describe and compare specific matchdays sessions and playing positions external load during congested fixtures in elite football when combining the objectives from two different training days in one session. Data was collected from 27 players from the 1^st^ Brazilian division (28.7 ± 18.61 years) that participated in the following training days during congested fixtures (n = 16 weeks): a) Match day+1 (MD+1^MD−2^, session 1 day after the game with tasks from MD+1 and MD-2 sessions, n = 9); b) Matchday-2 (MD-2^MD+2^, session 2 days prior to subsequent match with tasks from MD-2 and MD+2 sessions, n = 11); c) Matchday-1 (MD-1^MD+2^, session 1 day prior to subsequent match with tasks from MD-1 and MD+2 sessions, n = 12); and d) Matchday-1 (MD-1^MD+3^, session 1 day prior to subsequent match with tasks from MD-1 and MD+3 sessions, n = 11). External load was collected with global positioning systems, while internal load with the rating of perceived exertion (RPE). The MD+1^MD−2^ showed higher total distance covered (F = 116.92, p < 0.001) and player load (F = 56.67, p < 0.001) values than the other three training days, while both the MD+1^MD−2^ and MD-2^MD+2^ revealed higher distance covered at high-speed running (F = 22.43, p < 0.001) and high metabolic load distance covered (F = 75.98, p < 0.001) than both MD-1 sessions. Fullbacks covered higher high-speed running distance (F = 3.6, p = 0.033) than center backs, while midfielders reported higher RPE (F = 5.29, p = 0.003) values than defensive midfielders and fullbacks. Coaches may use the MD+1^MD−2^ to emphasize total distance covered, while both MD+1^MD−2^ and MD-2^MD+2^ to promote HSR and HML distance compared to MD-1 sessions. In addition, combining training sessions allows to normalize external load across playing positions with the exception of fullbacks that are exposed to higher stimulus.

## INTRODUCTION

A major aim during an association football training week (i.e., microcycle) is to enhance the team tactical coordination [1] while simultaneously maintaining high levels of physical fitness to face the increasing demands of the competition [2]. The design of a microcycle must entail daily training load fluctuations while decreasing the likelihood of inducing injuries and excessive fatigue [3]. These fluctuations are often identified throughout the microcycle. Accordingly, the first session of the week, often referred to as match day +1/+2 (MD, minus or plus; MD+1, i.e., training session held on the day after the competition) entails a double aim: i) recover the players that perform more than 60-min during the last match; ii) compensate load for those players’ that did not attain sufficient stimulus [4]. The MD-4 aims to develop the team intra-sectorial coordination by adopting intermittent small-sided games (SSG) in small spaces to emphasize acceleration and deceleration [5]. In contrast, training tasks involving a higher number of players and pitch space is used to promote team collective coordination while emphasizing total distance covered and distance covered at high-intensity [6, 7]. The MD-2 is focused on match strategical preparation by developing specific movement patterns and inter-sectorial coordination using training tasks in numerical superiority (e.g., 7 vs 4), while intending to emphasize sprinting distance [4, 6, 8]. Lastly, the MD-1 is often dedicated to promote the team offensive and defensive set pieces movement patterns during a low-intensity session [4].

Consequently, each MD emphasizes a specific team tactical aim, while adjusting the training tasks to induce appropriate stimulus. From a physical perspective, there is a trend towards increasing the training intensity from the first session of the week, often referred to as match day +1/+2 (MD, minus or plus; MD+1, i.e., training session held on the day after the competition) towards the MD-3 (i.e., training session held three days prior to the match) [2]. Meanwhile, a taper has been reported from MD-2 to MD-1 (i.e., training days held two and one days prior to the next match) to allow optimal performance in the match [5]. Considering a regular training microcycle (i.e., cycle composed by 5 training days: MD+1, MD-4, MD-3, MD-2 and MD-1 and one game per week), several studies have explored its external load distribution [9]. For example, Akenhead et al. [5] found that MD-4 presented higher mean load values for all variables than the remaining days, especially in terms of the number of accelerations and decelerations. Consequently, players have been observed to execute anywhere from approximately 110 to 150 accelerations (counted at > 3 m/s^−2^) and approximately 100 to 130 decelerations (counted at > 3 m/s^−2^), with midfielders reporting the highest numbers and strikers recording the lowest [4]. MD-3 also stands out as a session with a substantial load, often emphasizing total distance covered and distance covered at high-speed running (HSR) levels (> 21 km/h-1). In these sessions, players typically cover distances ranging from about 5100 m to 6500 m, with approximately 340 m of it categorized as HSR [4, 8]. Interestingly, during MD-3 in regular microcycles, fullbacks have been observed to cover more high-intensity distance than players in other positions, while midfielders tend to cover less high-intensity distance [4]. Furthermore, the majority of research has identified a tapering strategy in relation to MD-2 and MD-1. This tapering is evident in the reduced distance covered with high metabolic load (i.e., distance covered when metabolic power exceeds 25.5 W/kg), which averages around 405 m in MD-2 and approximately 580 m in MD-1, respectively [8].

Additionally, MD-2 places a strong emphasis on sprinting distance (> 25.2 km/h-1), often interspersed with periods of low-intensity activities. This reduction in load becomes even more pronounced in MD-1, where lower values are reported for most of the previously mentioned variables [4, 5, 8]. Despite these findings, differences in the load distribution have been found when analyzing teams from different cultures [10]. Thus, further research is required to provide additional information regarding general load values for each training day and its variability across teams in different continents.

Despite the increasing literature comparing different training days and playing positions’ external load during regular microcycles, the growing competitive demands in modern elite football [9] has promoted a change in the weekly periodization paradigm [8, 11]. In contemporary soccer, there is notable variability in the duration of microcycles, which can extend beyond 7 days (e.g., during breaks for FIFA National teams’ matches) or fewer than 3 days when teams engage in European competitions (e.g., the Champions League) or national cup fixtures. Consequently, microcycles can be categorized as ‘long’ when there is an interval of more than 8 days between matches within a week’s schedule, ‘regular’ when the gap between matches spans at least 7 days, or ‘short’ when teams contend with a mere 5 to 6 days between matches [8, 12]. When contrasting microcycle lengths (i.e., long, regular, and short), research findings have been inconsistent. For instance, some studies have reported higher external loads during longer microcycles compared to regular and short ones [13]. Conversely, when examining the number of games within a microcycle, Oliveira et al. [11] found no significant primary distinctions among weekly schedules featuring 3, 2, or 1 match, albeit they identified a noticeable trend towards reduced external load by the time of the last training session before a match (MD-1). Similarly, Anderson et al. [14] reported similar training load values when comparing regular with short microcycles. More recently, Lozano et al. [8] observed greater training volume and intensity, with the exception of accelerations, decelerations (measured at > 3 m/s^−2^), and HMLD, during longer microcycles in comparison to shorter and regular schedules [8]. These disparities among studies may arise from variations in training methodologies, coaching philosophies, and strategic preparations for upcoming matches [15].Furthermore, it is worth noting that while many studies investigating short-term schedules have considered a 5 to 6-day gap between matches, professional teams often face more demanding scenarios with as little as 2 to 3 days between competitive fixtures.

Short (i.e., three training days between matches) and very short (i.e., two training days between matches) cycles are now more prevalent, requiring different planning structures. For example, despite the adjustment in the total weekly training volume [11], coaches seem to maintain similar load prescriptions for the MD 2 and MD-1 sessions in congested fixtures (e.g., two matches in the same week) compared to regular weeks (i.e., only one weekly match) [16]. In fact, Swallow et al. [16] reported similar HSR (measured in distances higher than 19.8 km/h^−1^) distance in both MD-2 and MD-1 covered by semi-professional players during the two matches microcycles, despite identifying higher total distance and player load in the microcycles with 2 matches compared to the microcycles with 1 match. However, it is important to note that during the calendar with 3 games per week, professional teams trained 2 days between games without any day off. Therefore, in this case, the MD+1 also corresponded to the MD-2 by being 2 days before the subsequent match. These types of scenarios are emerging more often in the elite level, and thus further research is required to better support the technical staff on how to manage the players’ training loads properly. Advancing knowledge in this topic is urgent, as ~40% of players exposed to two to three matches within the same week have to complete all matches [17], increasing the injury risk [18]. To effectively address the fluctuating duration of training days within the microcycle, arising from competitive fixtures, a commonly employed strategy is to integrate objectives from two distinct days [8]. For instance, a training structure akin to MD-2, which prioritizes the refinement of team strategic movement patterns and highlights sprinting distances, can be combined with elements from MD+2 sessions. These MD+2 sessions are designed to facilitate low-intensity activities, aiding in the recovery of players who have exerted themselves for more than 60 minutes during a match, while also offering additional stimuli for players who did not experience such exertion. This amalgamation of training objectives may allow a balanced and effective approach to preparing the team for both physical recovery and match preparation. Despite such a strategy being applied to the acquisition days, less is known regarding its application to the first session after a match or to the last two before the following match. Therefore, the aim of the present study was to compare the external load profile of combining training sessions (MD+1 ^MD−2^, MD-2 ^MD+2^, MD-1 ^MD+2^ and MD-1 ^MD+3^) during very-short microcycles. A secondary aim of the study was to explore how these MD combinations affects the training load stimulus from different playing positions.

## MATERIALS AND METHODS

### Participants

Thirty-seven elite outfield football players from the Brazilian first division (i.e., Brasileirão – Série A) were selected based on a convenience sampling to participate in this study during the 2022 season (January to December). This club played in international competitions during the observation season (e.g., Copa Libertadores da América). This team is often forced to play several games within short periods of time (n = 77 matches during the season within the different competitions), which requires a vast squad to face such demanding contexts. In addition, the team had often to travel long distances to compete and play in very specific environmental conditions [19], that consequently require effective training-time strategies, such as combine two session structure in one session, between matches to maximize the team tactical preparation while avoiding inducing fatigue. This allowed to obtain the internal and external load from different microcycles employing the different MD combinations and playing positions. However, only 30 were retained for analyses (age: 28.7 ± 18.61 years; height: 180.75 ± 6.31 cm; body mass: 74.96 ± 6.98 kg; playing experience at a high level: 8.33 ± 5.66 years) on the basis that they attended the full training days for the selected weeks [5]. In turn, seven players were excluded as result of: (i) had fewer training days than required (less than 80% of the training days within the study duration) [6]; (ii) missing any session within the microcycle considered for analysis [12]; (iv) being in rehabilitation process both one-month before or during data collection [20]; (v) becoming injured or sick during more than two weeks during data collection; and (vi) did not complete at least 60-min of a competitive match [11]. Also, the goalkeepers (n = 5) were excluded from the present sample due to their specific and restrictive positioning, contributing to a distinct workload profile [16, 21]. The participating players comprised nine center backs, seven fullbacks, four defensive midfielders, four midfielders and six strikers (see table 1). The study protocol followed the recommendations of the Declaration of Helsinki; however, following previous guidelines regarding data collection in elite sports [22], ethics committee clearance was not required.

**TABLE 1:** Players’ characterization according to their playing positions.

Variables	Center Backs n = 9 Mean ± SD	Fullbacks n = 7 Mean ± SD	Defensive Midfielders n = 4 Mean ± SD	Midfielders n = 4 Mean ± SD	Strikers n = 6 Mean ± SD
Age (years)	25.8 ± 6.0	24.7 ± 6.2	25.5 ± 3.7	26.8 ± 9.7	25.0 ± 6.7
Height (cm)	188.3 ± 4.5	176.8 ± 2.6	178.5 ± 2.1	175.3 ± 3.2	179.2 ± 5.9
Weight (kg)	83.4 ± 6.4	70.4 ± 1.8	72.8 ± 2.2	70.0 ± 5.3	72.3 ± 4.0
Playing Experience (years)	9.1 ± 5.8	8.1 ± 5.2	8.5 ± 3.8	8.8 ± 9.7	7.0 ± 5.6

Note: cm = centimeters; kg = kilograms; n = number.

### Procedures

From a total of 45 recorded training weeks, only 16 were retained for analyses (see table 2). These 16 training microcycles were selected based on: i) belonging to a congested period (i.e., a maximum of three days between competitive matches); ii) there was a competitive match between training days without any day off between matches [14]; iii) being training days that combined two different objectives inside the training session. Hence, these 16 microcycles were thoughtfully chosen as they met several essential prerequisites, and all 30 participating players were available for these selected microcycles [14]. t’s worth noting that the training period also encompassed sessions structured with only a single-day design (e.g., MD-4); however, these sessions were intentionally excluded from our analysis. This decision was made to maintain a sharp focus on elucidating the effects of integrating two distinct day structures within a single training session. Our analyses exclusively considered the team’s primary training sessions, encompassing warm-up activities, the main training session phase, and the subsequent slowdown activities [11]. As a result, activities such as individual rehabilitation sessions or specialized individual training were not included in our analysis [11, 23, 24]. The training sessions themselves covered a wide spectrum, encompassing physical aspects (including warm-up routines and circuit-based activities), technical components (comprising rondos, ball control, passing exercises, and finishing exercises), and tactical elements (involving positional games, pressing tasks, specific movement patterns, and set pieces). All activities were monitored by the head coach and the technical staff without interference from the research team.

**TABLE 2:** Characteristics of training days.

Training Day	Typology	Number	Duration (min)	Description
MD+1^MD-2^	MD+1 / MD-2	9	50.3 ± 10.6	Session focused on low to moderate impact activities, composed by the players’ that performed less than 60-min of the match. The session MD+1 structure of the session consisted in high-intensity circuits, rondos, small-sided games. The MD-2 part of the session consisted in positional games and specific ball set plays as tapering exercises.

MD-2^MD+2^	MD+2 / MD-2	11	54.4 ± 11.3	Higher focus on technical-tactical preparation, such as match strategic plan. The MD+2 structure of the session consisted in specific rondos (e.g., 3+3+1 vs 3) and finishing drills under high speed and accelerations demands (e.g., 1 vs 1, 2 vs 1, 3 vs 2). The MD-2 profile of session was composed by offensive set movements, positional games with floaters and tactical exercises in numerical superiority.

MD-1^MD+2^	MD+2 / MD-1	12	49.5 ± 11.3	Higher focus on tactical preparation, such as working on set pieces. The MD+2 structure of the session consisted in ball control and passing tasks. The MD-1 structure of the session consisted in teambuilding activities and set pieces.

MD-1^MD+3^	MD+3 / MD-1	11	57.7 ± 10.3	Higher focus on tactical preparation, such as working on set pieces. The MD+3 structure of the session consisted in conditioning activation, followed by in rondos and pressing tasks. The MD-1 structure of the session consisted in teambuilding activities and set pieces.

Note: MD: match day; MD+1: 1 day after the match; MD+2: 2 days after the match; MD+3: 3 days after the match; MD-2: 2 days prior to the match; MD-1: 1 day prior to the match.

Training sessions during very short microcycles (i.e., three matches within one week) are often interspersed with two to three days, during which coaches typically employ the following strategies [8, 11, 14]: i) One session is dedicated to recovery, specifically designed for players who have participated for more than 60 minutes in the previous match. This session involves activities such as practicing passing patterns and employing team positioning in a slower manner to review specific errors made in the past match. Simultaneously, it includes moderate-intensity tasks for players who were not exposed to such stimulus, often involving SSG and positional games.; ii) the second session is focused on match strategic preparation, emphasizing defensive and offensive strategies. Coaches employ tasks that create a high superiority situation or incorporate passive opposition to reduce external load (this session is present when there are three training days between matches); iii) A set pieces session is conducted to refine specific movements for the upcoming match. These approaches leave limited time to develop the team’s tactical performance, which has been observed to be compromised during congested fixtures [25]. In response to these challenges, coaches have begun to adopt a mixed approach. This approach involves dedicating half of the session to recovery or tapering strategies to ensure that players are fresh for the upcoming match. In the remaining session time, coaches increase the intensity by incorporating the training structure of other training days to emphasize team movements and tactical preparation [8]. Considering the frequent exposure of the team to very short microcycles within the demanding competitive calendar, our study explores a combination of training sessions to address these challenges effectively (please refer to Table 2 for a detailed understanding of the training structure for each day): MD+1 MD−2 (session that was performed 1 day after the last match and with a distance of two days to the following match), MD-2 ^MD+2^ (2 days prior to the following match, and 2 days after the last one), MD-1 ^MD+2^ (1 day prior to the match and 2 days after the last one) and MD-1 ^MD+3^ (1 day prior to the match and 3 days after the last one) [5].

### Data Collection

Players’ external load during each training session was monitored using portable 10 Hz Global Positioning Systems (GPS, WIMU Pro^TM^ tracking systems, RealTrack Systems, Almeria, Spain). These units have been found to increase bias when capturing movements at high intensity (from ~1.6% bias during walking to ~3.5% bias during sprinting) and the type of movement (~1% for linear movements and ~1.6% for circular movements). Still, these systems have been found to be valid and reliable in capturing football players’ movement patterns and workload [26]. The GPS devices were activated 15 to 20-min before utilization, following the manufacturer’s recommendations. The number of satellites connected to devices was 10.6 0.3, with a horizontal dilution of precision of 0.9 ± 0.2. The players also wore the same unit to eliminate the inter-unit variability [27].

The players’ total distance covered in meters (m), high-speed running distance (HSR, above 21 km/h), player load (AU · min^−1^), and high-metabolic load distance (i.e., distance covered when the metabolic power higher than 25.5 W/kg) were used to characterize training days and playing positions’ external load. These variables have been used to compare different training days within the same or different microcycles [6, 8, 10, 16, 20, 28], and to compare different playing positions’ profiles over the training week [24].

The rating of perceived exertion (RPE) was collected 30 minutes after each training session using the CR-10 scale [29]. The individuals responded using a portable tablet, without the presence of other players. This procedure has been successfully reported to decrease pressure and biases related to the presence of peers [30]. Since players were familiar with this scale, no familiarization was required.

### Statistical Analysis

Descriptive data were presented as mean (M) ± standard deviation (SD). A linear mixed model was applied to compare the external load (distance covered, HSR, HMLD, Load) and internal load (RPE) according to the training days (MD+1^MD−2^, MD-2 ^MD+2^, MD-1 ^MD+2^, MD-1 ^MD+3^), MD-1), playing positions (fullback, center back, midfielder, striker) and the interaction between training days and playing positions. This statistical approach have been used to explore differences between training days within the microcycle [24], mainly from repeated measures data from players’ that possess different number of training days participation [31], while also taking into consideration fixed and random effects. Accordingly, the training day and playing positions were defined as categorical fixed effects, while the individual players’ and training session were considered as random effects. Additionally, the Bonferroni post-hoc was used for pairwise comparisons. Complementarily to the null hypothesis testing, Cohen’s d effect sizes (ES, 95% confidence intervals) were calculated for all comparisons using the following thresholds: < 0.2, trivial; 0.2 – 0.6, small; 0.6 – 1.2, moderate; 1.2 – 2.0, large; > 2.0 very large [32].

## RESULTS

**FIG. 1:**
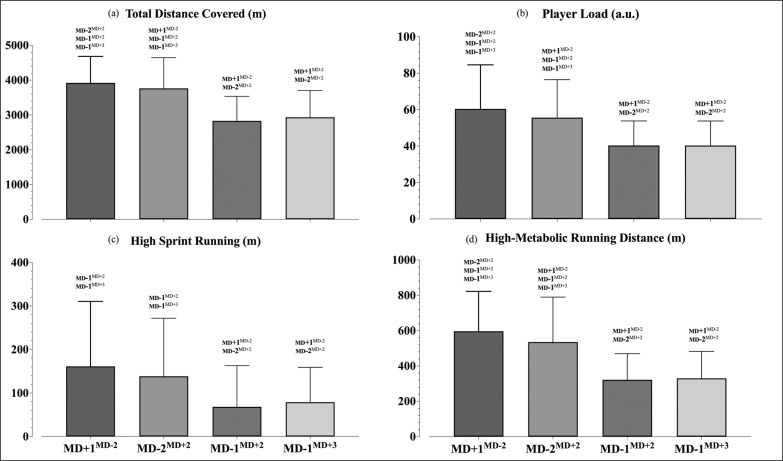
Descriptive (M ± SD) and inferential statistics from the training load data represented across four training days (MD+1^MD−2^, MD-2^MD+2^, MD-1^MD+2^ and MD-1^MD+3^). Note: having the training day above the bar means statistically significant difference between that day with that / those presented above.

**FIG. 2:**
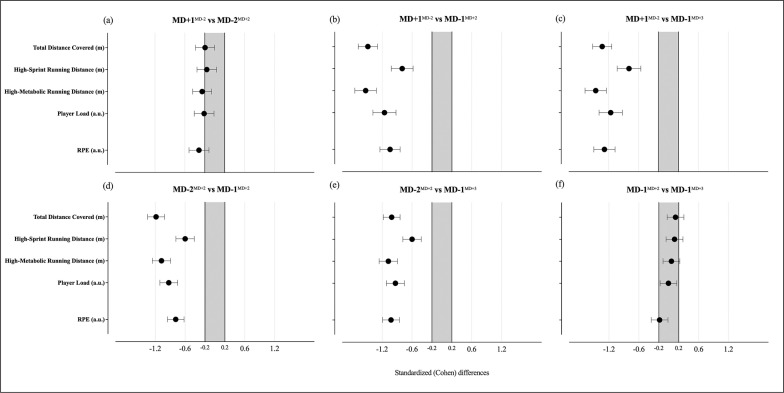
Standardized (Cohen) differences for the external and internal load according to the training. Errors bars indicate uncertainty in the true mean chances with 95% confidence intervals.

No significant effects or interactions were identified for the interaction between training days and playing positions (F = 0.93, p = 0.539). However, the linear mixed model identified several effects, mainly between training days. The differences between training days in relation to the MD are presented in Figure 1, Figure 2 and Table 3. The analysis revealed differences between the training days in all variables (total distance covered, F = 116.92, *p* < 0.001; high-sprint running, F = 22.43, *p* < 0.001; high-metabolic load distance covered F = 75.98, *p* < 0.001; load, F = 56.67, *p* < 0.001; and RPE, F = 40.49, *p* < 0.001).

**TABLE 3:** Descriptive statistics (M ± SD) from the external and internal load according to the training days in relation to the match day (MD).

Variables	MD+1^MD-2^	MD-2^MD+2^	MD-1^MD+2^	MD-1^MD+3^	Difference in Means (raw ± 95% CI)					

Mean ± SD	Mean ± SD	Mean ± SD	Mean ± SD	MD+1^MD-2^ vs MD-2^MD+2^	MD+1^MD-2^ Vs MD-1^MD+2^	MD+1^MD-2^ vs MD-1^MD+3^	MD-2^MD+2^ Vs MD-1^MD+2^	MD-2^MD+2^ Vs MD-1^MD+3^	MD-1^MD+2^ Vs MD-1^MD+3^
**External Loal**										
Total Distance Covered (m)	3907.2 ± 781.8	3763.2 ± 885.6	2828.7 ± 705.4	2927.5 ± 768.9	-159.4; ± 156.9	-1092.5; ± 143.0	-1010.4; ± 146.4	-933.2; ± 133.8	-851.1; ± 137.5	100.0; ± 124.2
Distance HSR (m)	160.3 ± 149.4	138.4 ± 133.4	68.1 ± 94.8	78.5 ± 80.4	-22.6; ± 27.8	-92.9; ± 25.5	-82.8; ± 24.9	-70.3; ± 19.3	-60.3; ± 18.6	10.1; ± 14.7
Distance HMLD (m)	593.5 ± 228.9	535.6 ± 254.6	322.3 ± 147.5	329.7 ± 152.4	-60.7; ± 45.9	-274.4; ± 38.8	-266.4; ± 39.2	-213.7; ± 34.8	-205.7; ± 35.4	8.0; ± 25.2
Player Load (a.u)	60.4 ± 24.2	55.6 ± 20.8	40.3 ± 13.5	40.3 ± 13.5	-4.8; ± 4.5	-20.0; ± 4.0	-20.1; ± 4.1	-15.2; ± 2.9	-15.3; ± 3.0	-0.1; ± 2.3

**Internal Loal**										
Rating of Perceived Exertion (a.u.)	4.5 ± 1.6	4.1 ± 1.4	3.0 ± 1.3	2.8 ± 1.1	-0.5; ± 0.3	-1.5; ± 0.3	-1.7; ± 0.3	-1.0; ± 0.2	-1.3; ± 0.2	-0.2; ± 0.2

Note: m = meters; HSR = High-sprint running; HMLD = High-Metabolic Load Distance; CI = Confidence Limits.

Accordingly, the MD+1^MD−2^ showed small to large higher distance covered compared with the remaining three conditions (*p* < 0.01, vs MD-2^MD+2^: ES with 95% CI: ES = -0.20 [-0.39; -0.00]; vs MD1^MD+2^: ES = -1.49 [-1.68; -1.29]; vs MD-1^MD+3^: ES = -1.33 [-1.52; -1.14]), followed by the MD-2^MD+2^ that presented moderate higher values than both the MD-1^MD+2^ (*p* < 0.01, ES = -1.18 [-1.35; -1.01]) and MD-1^MD+3^ (*p* < 0.01, ES = -1.01 [-1.18; -0.84]). As regards to the HSR, moderate higher values were found in the MD+1^MD−2^ when compared to both MD-1^MD+2^ (*p* < 0.01, ES = -0.8 [-1.02; -0.58]) and MD-1^MD+3^ (*p* < 0.01, ES = -0.79 [-1.03; -0.55]), as well as between MD-2^MD+2^ and both the MD-1^MD+2^ (*p* < 0.01, ES = -0.63 [-0.81; -0.46]) and MD-1^MD+3^ (*p* < 0.01, ES = -0.6 [-0.78; -0.41]). For the HMLD, results showed small higher values in MD+1^MD−2^ than in MD-2^MD+2^ (*p* = 0.027, ES = -0.25 [-0.44; -0.06]) and large higher than both MD-1^MD+2^ (*p* < 0.01, ES = -1.53 [-1.75; -1.31]) and MD-1^MD+3^ (*p* < 0.01, ES = -1.46 [-1.67; -1.24]), while the MD-2^MD+2^ also revealed moderate higher values than both MD-1 days (*p* < 0.01, vs MD-1^MD+2^, ES = -1.14 [-1.32; -0.95]; vs MD1^MD+3^, ES = -1.07 [-1.26; -0.89]). A small higher load was identified in MD+1^MD−2^ when compared to MD-2^MD+2^ (*p* = 0.04, ES = -0.21 [-0.41; -0.01]), while also a moderate higher than both the MD-1^MD+2^ (*p* < 0.01, ES = --1.15 [-1.39; -0.92]) and MD-1^MD+3^ (*p* < 0.01, ES = -1.16 [-1.39; -0.92]). In addition, the MD-2^MD+2^ also revealed moderate higher load values than the MD+1^MD+2^ (*p* < 0.01, ES = -0.93 [-1.11; -0.75]) and MD-1^MD+3^ (*p* < 0.01, ES = -0.93 [-1.11; -0.75]).

Lastly, the RPE values showed small to large higher values for MD-1^MD+2^ than the other training days (vs MD-2^MD+2^, *p* < 0.001, ES = -0.32 [-0.52; -0.11]; vs MD-1^MD+2,^ *p* < 0.01, ES = -1.04 [-1.24; -0.84]; and MD-1^MD+3^, *p* < 0.01, ES = -1.28 [-1.5; -1.07]), while also moderate higher in MD-2^MD+2^ than in MD-1 sessions (*p* < 0.001, vs MD-1^MD+2^, ES = -0.79 [-0.95; -0.62]; vs MD-1^MD+3^, ES = -1.02 [-1.19; -0.85]).

**FIG. 3:**
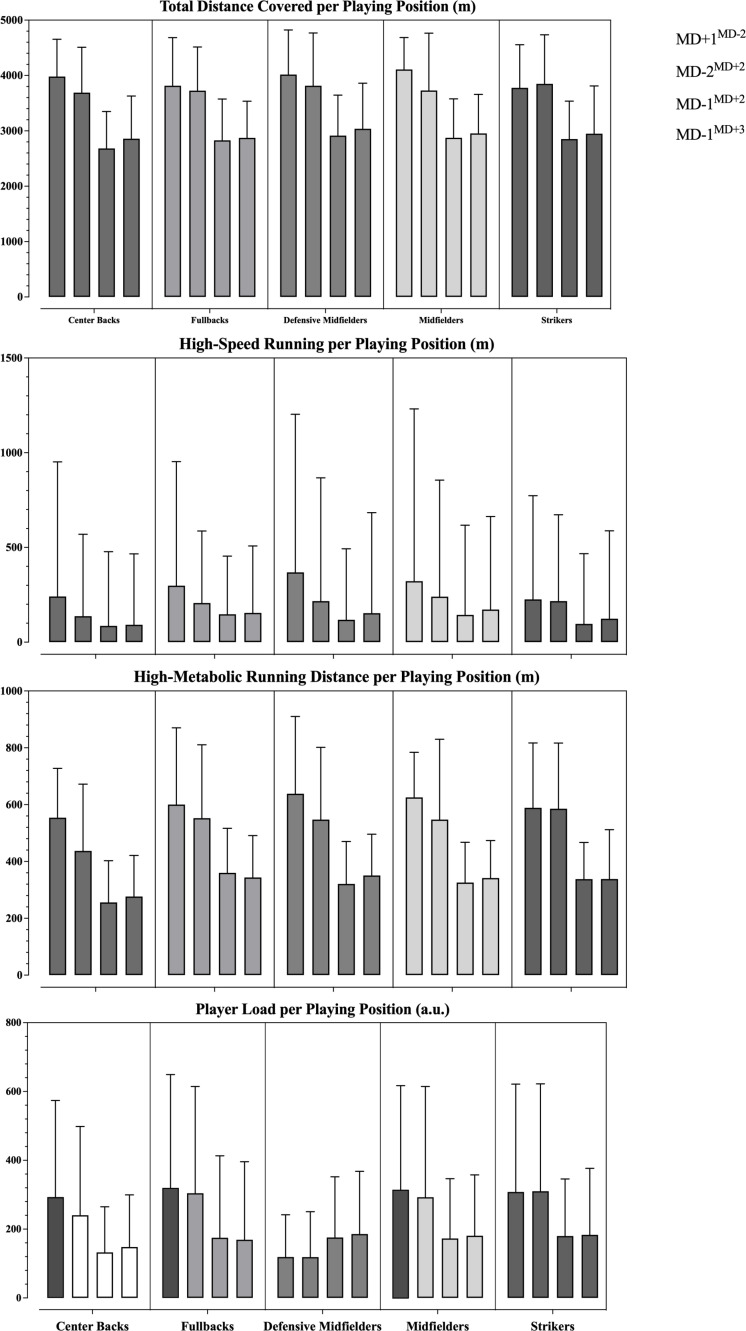
Descriptive statistics (M ± SD) from the training load data represented across four training days (MD+1^MD−2^, MD-2^MD+2^, MD-1^MD+2^ and MD-1^MD+3^) according to the playing positions.

The differences between training days in relation to the MD are presented on Figure 3 and Table 4. In general, no effects were identified between playing positions for the studied variables. Differences were only identified for the high-speed running distance (F = 3.6, p = 0.033) and the RPE (F = 5.29, p = 0.003). In this respect, the fullbacks covered more high-speed running distance compared to the center backs (p = 0.002). The midfielders reported higher RPE values than both the defensive midfielders (p = 0.019) and fullbacks (p = 0.007).

**TABLE 4:** Descriptive statistics (M ± SD) from the external and internal load according to playing positions in each training session.

Variables	Difference in means (raw ± 95% CI)									

	Centre Back vs				Fullbacks vs			Defensive Mid.vs		Midfielders vs.

	Fullbacks	Def. Mid.	Midfielders	Strikers	Def. Mid.	Midfielders	Strikers	Midfielders	Strikers	Strikers
MD+1^MD−2^										
Total Distance Covered (m)	-167.1; ± 361.8	35.2; ± 403.3	127.4; ± 328.5	-205.0; ± 330.0	202.3; ± 435.2	294.5; ± 368.0	-37.9; ± 369.7	92.2; ± 408.7	-332.4; ± 337.1	-240.2; ± 410.3
Distance HSR (m)	73.7; ± 70.0	82.4; ± 89.1	17.6; ± 59.6	22.1; ± 50.9	8.6; ± 100.8	-56.1; ± 76.8	-51.6; ± 70.7	-64.8; ± 94.2	-60.2; ± 89.7	4.5; ± 60.3
Distance HMLD (m)	46.4; ± 105.3	84.4; ± 127.4	71.4; ± 88.2	34.9; ± 90.0	294.5; ± 120.1	281.6; ± 76.6	-11.4; ± 111.0	-12.9; ± 130.5	-49.4; ± 131.9	-36.5; ± 94.9
Load (a.u)	12.3; ± 15.1	2.9; ± 6.9	0.7; ± 5.4	-2.8; ± 4.8	-9.5; ± 15.8	-11.6; ± 15.3	-15.2; ± 15.1	-2.1; ± 7.2	-5.7; ± 6.8	-3.6; ± 5.3
Rating of Perceived Exertion (a.u.)	-0.7; ± 0.8	-0.8; ± 0.9	0.6; ± 0.8	-0.0; ± 0.8	-0.1; ± 0.8	1.3; ± 0.6	0.7; ± 0.7	1.4; ± 0.8	0.7; ± 0.8	-0.7; ± 0.7

MD-2^MD+2^										
Total Distance Covered (m)	35.6; ± 293.5	124.8; ± 352.6	39.1; ± 377.6	158.8; ± 308.6	89.2; ± 338.7	3.4; ± 364.8	123.2; ± 292.3	-85.7; ± 412.7	34.0; ± 351.7	119.8; ± 376.8
Distance HSR (m)	82.3; ± 49.3	42.4; ± 42.8	70.4; ± 50.4	81.6; ± 43.7	-39.9; ± 49.3	-11.9; ± 56.0	-0.6; ± 50.0	28.0; ± 50.5	39.3; ± 43.7	11.2; ± 51.2
Distance HMLD (m)	115.3; ± 89.6	110.1; ± 96.9	110.2; ± 104.9	148.6; ± 84.6	-5.2; ± 97.6	-5.2; ± 105.5	33.2; ± 85.4	0.0; ± 111.6	38.4; ± 93.1	38.4; ± 101.4
Load (a.u)	11.3; ± 9.3	4.9; ± 5.0	1.7; ± 4.9	2.01; ± 4.3	-6.4; ± 9.8	-9.6; ± 9.7	-9.3; ± 9.4	-3.2; ± 5.8	-2.9; ± 5.2	0.3; ± 5.2
Rating of Perceived Exertion (a.u.)	-0.4; ± 0.5	-0.6; ± 0.5	0.3; ± 0.6	0.2; ± 0.5	-0.1; ± 0.4	0.7; ± 0.5	0.6; ± 0.4	0.9; ± 0.6	0.7; ± 0.4	-0.1; ± 0.6

MD-1^MD+2^										
Total Distance Covered (m)	146.2; ± 259.9	231.4; ± 274.4	192.4; ± 279.2	169.1; ± 239.0	85.2; ± 279.7	46.2; ± 284.3	22.9; ± 245.0	-39.0; ± 297.5	-62.3; ± 260.6	-23.2; ± 265.6
Distance HSR (m)	77.9; ± 39.1	32.9; ± 33.7	40.4; ± 32.1	20.0; ± 26.8	-45.4; ± 41.1	-38.8; ± 40.1	-57.9; ± 35.9	6.7; ± 34.9	-12.5; ± 29.8	-19.2; ± 28.4
Distance HMLD (m)	104.2; ± 55.9	65.4; ± 58.0	70.1; ± 58.5	82.3; ± 49.5	-38.7; ± 49.7	-34.1; ± 58.3	-22.0; ± 49.3	4.8; ± 60.3	16.9; ± 51.7	12.2; ± 52.3
Load (a.u)	8.1; ± 5.9	5.4; ± 4.3	2.5; ± 3.6	2.6; ± 3.1	-2.7; ± 6.5	-5.7; ± 6.0	-5.5; ± 5.8	-2.9; ± 4.5	-2.8; ± 4.1	0.2; ± 3.4
Rating of Perceived Exertion (a.u.)	-0.3; ± 0.5	-0.5; ± 0.5	-0.1; ± 0.6	0.1; ± 0.5	-0.2; ± 0.5	0.2; ± 0.5	0.4; ± 0.4	0.4; ± 0.6	0.6; ± 0.5	0.2; ± 0.5

MD-1^MD+3^										
Total Distance Covered (m)	-7.8; ± 257.8	178.5; ± 317.2	95.4; ± 302.7	90.5; ± 298.2	164.1; ± 291.0	81.1; ± 275.0	76.1; ± 269.9	-83.1; ± 320.1	-88.0; ± 316.0	-4.9; ± 301.4
Distance HSR (m)	69.7; ± 29.3	36.7; ± 26.0	57.4; ± 34.0	26.6; ± 20.8	-33.0; ± 32.3	-12.3; ± 39.0	-43.2; ± 28.4	20.8; ± 36.6	-10.1; ± 25.1	-30.9; ± 33.4
Distance HMLD (m)	67.0; ± 54.8	73. 9; ± 57.5	65.1; ± 56.7	61.6; ± 58.0	6.9; ± 56.1	-1.9; ± 55.2	-5.4; ± 56.6	-7.7; ± 57.4	-11.2; ± 58.7	-3.5; ± 58.4
Load (a.u)	4.9; ± 5.5	3.3; ± 4.7	0.1; ± 4.1	1.0; ± 4.1	-1.6; ± 6.1	-4.9; ± 5.6	-3.9; ± 5.6	-3.3; ± 4.8	-2.4; ± 4.8	0.9; ± 4.2
Rating of Perceived Exertion (a.u.)	0.1; ± 0.4	-0.3; ± 0.5	0.4; ± 0.5	0.3; ± 0.4	-0.4; ± 0.4	0.3; ± 0.4	0.2; ± 0.4	0.7; ± 0.5	0.6; ± 0.4	-0.2; ± 0.4

Note: m = meters; HSR = High-sprint running; HMLD = High-Metabolic Load Distance; CI = Confidence Limits.

## DISCUSSION

This study aimed to describe the external load during congested fixtures in elite football when combining the objectives from two different training days in one session. Overall, the results highlight that: 1) combining MD+1 and MD-2 training content resulted in higher total distance covered, compared to the other conditions, 2) HSR and HMLD distance were higher in MD+1^MD−2^ and MD-2^MD+2^, compared to both MD-1 sessions, and 3) training loads were generally evenly distributed between playing positions, with the exceptions of the fullbacks, that covered more HSR distance compared to the center-backs, and the midfield players, that reported higher RPE values than the defensive midfielders and fullbacks.

The fact is that the increasing competitive trends in modern elite football will likely make these shorter microcycles the most common standard since top teams are constantly playing 2–3 games in a single week [9], which results in short and very short microcycles (3 and 2 days between 2 matches, respectively) [8, 11]. Planning this type of short period is often a challenge for the technical staff since there might not be enough time for players to fully recover from the previous match, while coaches must prepare the team for the next one. Furthermore, previous investigations [25] have unequivocally showcased the unwavering physical prowess displayed by athletes in the crucible of congested fixtures. However, a conspicuous deviation from this norm comes to light as we observe a discernible decline in players’ movement synchronization [25]. This decline raises the tantalizing possibility of a correlation with the constrained temporal window for formulating essential tactical and strategic frameworks crucial in countering the imminent opposition. Consequently, it seems tenable to propose that coaches may progressively place a heightened emphasis on fortifying team tactical preparation during periods characterized by fixture congestion [33]. To face such competitive requirements, coaches may combine the training structure of two different training days aiming to promote enough stimulus to enhance the team’s tactical behavior while adjusting the load to avoid the accumulation of fatigue [8]. While this strategy may sound appropriate to prepare the teams, scientific evidence is scarce in providing descriptive results on the effect of combining two different days’ structures within the same training session. Nonetheless, the repercussions of these strategies on players’ external load remain uncharted territory.

Depending on the number of sessions between games, the first and/or the second session are often the most physically demanding, while a clear taper has been identified in the MD-1 [11]. In fact, Oliveira et al. [11] compared training days from weeks with 1, 2 or 3 matches and identified the MD+1 as the most physically demanding session, with statistically significant effects compared to the MD-2 and MD-1. The results from this study seem to support this trend, as players covered more total distance, HSR, HMLD distance and also presented higher Player Load in the first two training days, i.e., MD+1^MD−2^ and MD-2 ^MD+2^, than in the session that preceded the match (MD-1^MD+2^ or MD-1^MD+3^), independently of the structure adopted. In contrast to these findings, Swallow et al. [16] found higher total distance and player load in the MD-1 when having 2 games per week than when having just 1 game. However, these differences may rely on the differences between the playing levels (i.e., Swallow’s study explored the training loads in semi-professional players that had only a maximum of 3 training days per week, while in this study the participants were professional players) or GPS units used (i.e., PlayerTek vs Wimu). Therefore, during congested fixtures, and independently of combining the training structure of two training days within the same session or following just one day structure, coaches seem to design training tasks that are more physically demanding in the first two sessions of the week and then decrease it in the last one.

The MD+1^MD−2^ group also exhibited notable distinctions, displaying greater metrics in terms of total distance covered, high metabolic load distance (HMLD), and overall player load when compared to the MD-2 MD+2 group. The MD+1 phase typically focuses on the recuperation of players who have participated for more than 60 minutes in a match, involving specialized recovery-oriented sessions. In contrast, those players not exposed to such extended match periods confront sessions that rank among the most demanding within the microcycle [4]. However, it is important to note that in the present study, data from the MD+1^MD−2^ group excluded players who had undergone the recovery session, a deliberate exclusion designed to reduce data variability [11]. The training sessions for players receiving physical stimulus in the MD+1 phase encompassed a blend of high-intensity circuits, rondos, and small-sided games (SSG), as well as positional games and passing patterns. Such training methodologies appear to accentuate players’ capacity for acceleration and deceleration, notably observed during SSG, circuits, and rondos, while the emphasis on distance covered becomes evident during positional games and passing patterns [6, 12]. Given that the primary objective is to replicate the intensity experienced during 60 minutes of a match, it is reasonable to anticipate that the MD+1^MD−2^ group would exhibit higher performance values in comparison to the MD-2 group, where a tapering strategy is employed [2, 11, 14]. While it is expected that a decrease in training load in days closer to the match, it is also expected that during short length cycles, coaches decrease the load in the MD-2, which often refers to the second training session in a congested period. It is known that depending on the markers considered (i.e., creatine kinase, jumping ability, blood sample), most of them return to baseline values only between 48 h to 72 h after the match [34]. Therefore, under congested fixtures, coaches seem to decrease the training load in the MD-2 as a result of the proximity to subsequent match [9, 11], but also taking into consideration the recovery process of line-up players, as well as the load that substitutes and non-used players were exposed on the previous training (i.e., MD-1). The lack of effects for the HSR distance would be somehow expected. That is, the MD+1 focus on providing load to the substitute and non-used players, who often are in a lower number than the remaining sessions. In this respect, it is well known that a lower number of players on a game-based task combined with a lower pitch size leads to lower distance covered at high-intensity [35], while the MD-2 session has the participation of the lineup players that are not fully recovered and thus, coaches often avoid creating very high-intensity stimulus [3].

Following the MD+1^MD−2^ session, it was evident that the MD2^MD+2^ session imposed a more substantial training load than the cumulative MD-1 days. In conventional training microcycles, coaches frequently employ this session to cultivate strategic movement patterns based on an analysis of the opposition. For instance, they may simulate specific scenarios, such as goal kick build-ups, based on video analysis of the opposition’s pressure. This session also incorporates training tasks designed to enhance sprinting abilities, such as analytical drills and finishing drills based on long-distance passes. It culminates in a match simulation (typically involving a Gk+10 v 10+Gk). In this simulation, the defensive team is often confined to specific areas to reduce physical load while allowing for a review of team tactical coordination. Research has revealed that such sessions generally lead to players covering distances ranging from approximately 3490 to 5780 meters [4, 5], with high-speed running (HSR) segments varying from around 23 (measured above 21 km/h^−1^) [5] to 284 m (measured above > 19.8 km/h^−1^) [16]. Additionally, players tend to accumulate a player load of approximately 543 a.u. [5].

In shorter-term microcycles, studies have shown that players cover distances ranging from about 4780 [8] to 6700 m [11], with HSR segments (measured above 19 km/h) ranging from approximately (measured above > 21 km/h^−1^) [8] to 280 meters (measured above > 19 km/h^−1^) [11]. These sessions also typically result in players covering approximately 760 meters of HMLD and reporting player load values of approximately 70 a.u. [8].

While prior research has suggested similar training load values in MD-2 sessions across regular and short microcycles [8], a combined approach seems to elicit a distinct training stimulus profile. In the combined MD-2^MD+2^ session, players covered approximately 3760 meters in total distance, with approximately 140 meters at high-intensity (measured above 21 km/h), around 535 meters of HMLD, and a player load of about 55 a.u. It’s worth noting that these values are lower than the upper limits observed in typical microcycles, particularly in terms of total distance covered, despite comparable HSR figures. These differences may be attributed to session variations, as the study by Oliveira [11] categorized the first session as MD-2 rather than MD+1 when only two sessions were considered. Additionally, disparities in data collection technology (WIMU vs. DatatraX®) and variations in metric parameters, such as HSR thresholds (above 19 km/h, above 19.8 km/h, and above 21 km/h), could contribute to these differences. Furthermore, the microcycle length differs between studies, with a 3-day gap between matches in the present study and a 5-day gap in the Oliva-Lozano study [8], which can limit direct comparisons.

In general, adopting a combined session structure like MD-2^MD+2^ appears to induce lower total distance covered values compared to the majority of typical and short microcycles, despite similar HSR figures. Consequently, this combined approach may be advantageous for enhancing player recovery from the preceding match, aiding in the removal of blood lactate and pH recovery [3] while still addressing the speed development essential for this training session [6]. Additionally, this approach seems to alleviate the mechanical load on players, as indicated by lower HMLD and player load figures, which could enhance player readiness for upcoming matches.

The final training session preceding a match, often referred to as MD-1, is typically dedicated to refining team set pieces and reviewing strategic movements, such as strategies for pressing the opposition during their build-up. Given its proximity to the match, this session is usually shorter in duration and consists of two to three key exercises. These exercises often include activities like rondos for specific player activation, interspersed with reaction speed drills, a 10 vs 10 simulation of passive strategic movements, and concludes with offensive and defensive set piece drills. Available data has generally shown consistent external load characteristics during MD-1, regardless of the length of the microcycle [8]. For instance, in the study by Oliva-Lozano et al. [8]., similar metrics were reported for distance covered (approximately 4091 m vs. 3950 m), high-speed running (measured above 21 km/h, approximately 130 m vs. 127 m), high metabolic load distance (approximately 611 m vs. 581 m), and player load (approximately 58 a.u. vs. 56 a.u.) when comparing short microcycles (5 days between matches) with regular microcycles (6 days between matches). When considering the number of matches within a week, higher total distance covered was found during the microcycle with two matches (with two sessions between matches, approximately 5100 m) compared to one match (with three sessions between matches, approximately 4423 m), along with higher player load (approximately 250 a.u. vs. 220 a.u.) [16]. Additionally, Oliveira et al. [11] reported total distance covered values ranging from approximately 3620 m to 4420 m and RPE values of around 3 a.u. when facing three matches within the same week with two training sessions in between.

In the present study, MD-1 sessions (both MD-1^MD+2^ and MD1^MD+3^) reported lower values for total distance covered (approximately 2830 to 2930 m), high-speed running (approximately 68 to 79 m), high metabolic load distance (approximately 322 to 330 m), and player load (approximately 40 a.u.) compared to previous research. However, it’s important to note significant methodological differences, as distance between matches varied from 5 days [8] to 2 days with [16] or without day off [11], while the present study considered 3 sessions between matches. These discrepancies make direct comparisons challenging, despite the clear indication of lower external load when employing a two-session structure. Interestingly, no significant differences were observed when comparing the two MD-1 structures (MD-1^MD+2^ and MD-1^MD+3^). This lack of distinction could be attributed to the similar activity profiles demanded by the tasks in these different structures. For instance, in MD-1^MD+2^, players engaged in ball control and passing drills, while in MD-1^MD+3^, they participated in rondos and passive pressing tasks against opposition. Ball control and passing drills are typically of low-intensity effort and were applied for a short duration, contributing to a similar physical demand when compared to rondos and passive pressing tasks. Moreover, this MD-1 session is known for its lower volume [4], so it’s plausible that the limited exposure time to various tasks, coupled with consistent exposure to set pieces, resulted in the lack of discernible differences. Nevertheless, coaches may intentionally introduce variability into the training structure during MD-1 to prevent monotony and align the training content with the analysis of the upcoming opposition. For example, MD-1^MD+2^ could be used to refine offensive passing patterns when the team is expected to dominate possession, while MD-1^MD+3^ might be more appropriate when facing higher-quality opponents and expecting to spend more time on defensive tasks. This adaptability allows coaches to tailor their training to the specific demands of the upcoming match and optimize player preparation.

At last, minimal differences were found in the training load exposure between playing positions. It is known that the match demands vary depending on the positional role that players have on the field. For instance, center backs usually cover lower total distance and HSR than fullbacks, central-midfielders, wide-midfielders and forwards [36]. Wide midfielders and forwards cover greater total distances, while wide-midfielders and second attackers present higher peak match speeds and frequency of high-intensity activities [36]. Since soccer training is very game based, i.e., exercises usually try to replicate the match demands and the different phases of the match (i.e., defensive organization, offensive transition, offensive organization), these between playing position differences are also observed in training. Akenhead et al. [5] found that center midfielders covered higher total distance when compared to center backs during the microcycles’. Other authors also found that fullbacks covered a higher total distance than the remaining positions, specifically on the MD-4 and MD-3 [4]. These small differences were somewhat expected and can be attributed to the overall characteristics of the sessions explored in our study (i.e., rondos, circuits, set pieces, positioning exercises, and overall smaller spaces), common in lower loads training days [4, 6, 8]. Between positions differences are more commonly observed in training days with higher loads, such as MD-4 and MD-3, which are characterized by larger playing areas that try to replicate the match demands [4, 5]. When looking into the days characterized by lower training loads (MD+1, MD-2, and MD-1), the results of other studies [5, 6] generally align with ours. The tactical role of a player is known to be a powerful determinant of their match physical performance, so it is necessary that the conditioning stimulus of training has a positional element to it [37]. However, the focus of the first and last sessions of the microcycle is not on the conditioning side, but more on the match compensation (in case of substitutes) or recovery, technical-tactical elements, activations and set pieces, respectively [4, 5, 8].

In summary, the integration of training structures from various days offers a valuable means to reduce external load when compared to the more conventional microcycles or shorter-term microcycles that typically feature only one training structure. Given that the primary disparities in player performance during congested fixture periods are closely linked to tactical behaviors, coaches can strategically employ these combined training structures to emphasize and refine these tactical aspects while concurrently mitigating the overall external load. This approach allows for a more balanced and position-specific normalization of the training stimulus across the team, ultimately contributing to enhanced player preparation and performance in congested fixture scenarios.

### Limitations and future directions

The study has some general limitations. Firstly, it’s important to note that our study focused exclusively on one elite football team. Thus, it inherently limits the generalizability of our findings to a broader spectrum of elite football teams. Given the inherent diversity in coaching philosophies, player characteristics, and playing styles across different teams, it’s crucial to recognize that training load strategies may vary significantly. Furthermore, we employed convenience sampling in this study for practical reasons. However, it’s crucial to acknowledge that convenience sampling introduces potential bias into the sample selection process, as participants are chosen based on their accessibility and availability. Consequently, it is imperative to interpret the findings of this study within the context of this nonrandom sampling method. Caution should be exercised when attempting to generalize our results to broader populations of elite football players. To address these limitations, future research endeavors should consider employing more extensive and diverse participant recruitment methods. This approach would likely provide a more comprehensive understanding of the topic, ensuring a broader representation of elite football players. Additionally, the data presented in our study is primarily descriptive in nature. Consequently, it would be important that further studies compare training sessions with only one structure with those presenting structures from two different training days. Such comparative analyses would help uncover the potential advantages of combining different training structures within a single session, offering deeper insights into the underlying mechanisms and causality behind our findings.

## CONCLUSIONS

Overall, the results from this study highlighted that combining two different training days in the same session contributed to a higher training load in the MD+1^MD−2^, followed by MD-2^MD+2^ and then by both MD-1^MD+2^ and MD-1^MD+3^. As regard to players positioning, adopting combined sessions led to a similar profile between players, apart from fullbacks that covered more HSR distance than the center backs. In this respect, combining sessions that may stress the players’ physical performance more with sessions that are more focused on taper (e.g., MD-1 with MD+2; MD-2 with MD+2; MD-1 with MD+2; or MD-1 with MD+3) seems to level the training load across positions. Thus, combining two different training structures within the same session may help coaches best tailor the training loads according to the tactical preparation requirements and regulate playing positioning stimulus. However, to properly understand the potential of this strategy, further studies may compare the composed by the structure of 2 different training days to sessions based only on one training day.
